# Serological Evidence of Hantavirus in Bats from the Brazilian Atlantic Forest: An Investigation of Seroreactivity and Cross-Reactivity of Neotropical Bat Samples Using Nucleoproteins of Rodent- and Bat-Borne Hantaviruses

**DOI:** 10.3390/v16121857

**Published:** 2024-11-29

**Authors:** Caroline Lacorte Rangel, Silvia da Silva Fontes, Marcus Vinicius de Mattos Silva, Jorlan Fernandes, Janaina Figueira Mansur, Emmanuel Messias Vilar, Sócrates Fraga da Costa-Neto, Roberto Leonan Morim Novaes, Pedro Cordeiro-Estrela, Ricardo Moratelli, Elba Regina Sampaio de Lemos, Ronaldo Mohana Borges, Rodrigo Nunes Rodrigues-da-Silva, Renata Carvalho de Oliveira

**Affiliations:** 1 Laboratório de Hantaviroses e Rickettsioses, Instituto Oswaldo Cruz, Fiocruz, Rio de Janeiro 21040-900, Brazil; carolinellacorte@gmail.com (C.L.R.); silviasilvafontes@gmail.com (S.d.S.F.); jorlan@ioc.fiocruz.br (J.F.); elemos@ioc.fiocruz.br (E.R.S.d.L.); 2 Laboratório de Genômica Estrutural, Instituto de Biofísica Carlos Chagas Filho, Universidade Federal do Rio de Janeiro, Rio de Janeiro 21941-971, Brazil; marcus.mattos@hotmail.com (M.V.d.M.S.); janaina.mansur@bio.fiocruz.br (J.F.M.); mohana@biof.ufrj.br (R.M.B.); 3 Faculdade de Ciências Biológicas e Saúde, Universidade do Estado do Rio de Janeiro, Rio de Janeiro 23070-200, Brazil; 4 Laboratório de Mamíferos, Departamento de Sistemática e Ecologia, CCEN, Universidade Federal da Paraíba, João Pessoa 58051-900, Brazil; messiashp@gmail.com (E.M.V.); pedrocometa@gmail.com (P.C.-E.); 5 Fiocruz Mata Atlântica, Fundação Oswaldo Cruz, Rio de Janeiro 22713-375, Brazil; socratesfcneto@gmail.com (S.F.d.C.-N.); robertoleonan@gmail.com (R.L.M.N.); rimoratelli@gmail.com (R.M.)

**Keywords:** bat-borne virus, seroprevalence, Chiroptera, tropical rainforest, recombinant nucleoproteins, ELISA

## Abstract

Hantaviruses are zoonotic pathogens associated with severe human diseases such as hemorrhagic fever with renal syndrome and hantavirus pulmonary syndrome. Despite the extensive study of rodent-borne hantaviruses, research on bat-associated hantaviruses remains limited. This study aimed to investigate the seroprevalence and cross-reactivity of neotropical bat samples with rodent- and bat-associated recombinant hantavirus nucleoproteins (rNPs) to improve hantavirus surveillance in the Brazilian Atlantic Forest. The studied bat population consisted of 336 blood samples collected over nearly a decade in five Brazilian states (Bahia, Rio de Janeiro, Santa Catarina, Paraná, and Minas Gerais). Antibodies were detected using IgG ELISA assays with rNPs from bat-borne *Mobatvirus xuansonense* (XSV) and *Loanvirus brunaense* (BRNV) and the rodent-borne hantaviruses *Orthohantavirus andesense* (ANDV) and *Orthohantavirus seoulense* (SEOV). Results indicated a higher seroprevalence for the BRNV rNP (36.6%) compared to ANDV (7.4%), SEOV (5.7%), and XSV (0.6%). The high sensitivity of the BRNV rNP and the cross-reactivity observed with the ANDV rNP, the main protein used for serological tests in the Americas, indicates that BRNV rNP is a better antigen for the accurate detection of antibodies against hantaviruses in Brazilian bats. These findings underscore the presence of unknown hantaviruses antigenically similar to BRNV in Brazilian bat populations and highlight the urgent need for identifying better antigens for comprehensive hantavirus monitoring in bats.

## 1. Introduction

Mammals play a critical role in maintaining zoonotic sylvatic cycles, with bats (order Chiroptera) standing out as major contributors [1]. Bats serve as natural reservoirs for a wide range of emerging zoonotic pathogens, particularly viruses such as those that are the causative agents of rabies (*Rhabdoviridae*), influenza (*Orthomyxoviridae*), Ebola, Marburg (*Filoviridae*), Nipah, Hendra (*Paramyxoviridae*), SARS (*Coronaviridae*), and renal and pulmonary syndromes (*Hantaviridae*). The diversity of generalist bat species capable of adapting to anthropized environments, their ability to occupy a variety of ecological niches, long lifespan, coloniality, wide geographic ranges, high dispersal ability, and the diversity of trophic guilds of bats support their potential role in the global spread of zoonotic diseases [2].

In recent years, numerous studies have been conducted to investigate viral pathogens in bats [1,3]. Among these, hantaviruses are important (re)emerging zoonotic pathogens classified in the order *Elliovirales*, in the *Hantaviridae* family [4]. Hantavirus transmission occurs most commonly by inhalation of the aerosolized virus particles contained in the dried urine, feces, and saliva of infected animals; it has been found to cause clinically mild, moderate, or severe disease in humans, including hemorrhagic fever with renal syndrome (HFRS) in Asia, Europe and Africa and hantavirus pulmonary syndrome (HPS) throughout the Americas [5]. While all human cases are attributed to rodent-borne orthohantaviruses, attention to bat-borne hantaviruses has steadily increased [6]. Currently, there are two genera (*Mobatvirus* and *Loanvirus*) comprising the following seven bat-borne hantavirus species: *Mobatvirus laibiense* (LAIV); *Mobatvirus xuansonense* (XSV); *Mobatvirus quezonense*; *Mobatvirus dakrongense*; *Mobatvirus robinaense*; *Loanvirus longquanense*; and *Loanvirus brunaense* (BRNV). These are officially recognized by the International Committee on the Taxonomy of Viruses [7,8,9]. However, information regarding bat-borne hantavirus species, their distribution, and hosts remains limited, highlighting the need to develop novel and effective diagnostic tools for bat-borne hantavirus surveillance [10].

The diagnosis of hantaviruses primarily relies on serological tests, detecting specific class G antibodies against the hantavirus nucleoprotein (NP) in enzyme-linked immunosorbent assays (ELISA) [6,11]. This protein elicits an early, robust, and sustained humoral response, allowing for the early detection of exposure [12,13,14]. This approach has been highly effective in the diagnosis of human cases and eco-epidemiologic studies, particularly in the detection of IgG antibodies in both human and animal reservoirs [11,15]. However, the surveillance of bat-borne hantaviruses is scarce. In southwestern China, serologic testing of bat samples with the nucleoproteins of LAIV, XSV, and *Orthohantavirus seoulense* (SEOV) has been conducted [16]. Brazilian studies using the nucleoprotein of Araraquara virus (ARAV), an *Orthohantavirus andesense* (ANDV) variant and rodent-borne virus, have demonstrated the circulation of hantaviruses in bats [17,18,19]. However, due to antigenic differences between NP from different hantaviruses, there is no “gold standard” protein for bat surveillance.

Recently, an in silico evaluation of the conservation of B cell epitopes between the NP of bat- and rodent-borne hantaviruses suggested a low cross-reactivity between antibodies against the NP of bat-borne hantaviruses and the proteins of rodent-borne hantaviruses [10]. These data, combined with the lack of bat-borne hantavirus information in the Americas, underscore the need for more accurate serological testing to provide a true picture of the prevalence of bat-borne hantaviruses, their geographic distribution, and to help to understand the dynamics of virus–host interactions. In this context, this study aimed to investigate the serological evidence of bat-borne hantaviruses in samples from bats from the Brazilian Atlantic Rainforest biome, using the recombinant NP (rNP) of the bat-borne hantaviruses, XSV and BRNV, and the rodent-borne hantaviruses, ANDV and SEOV.

## 2. Materials and Methods

### 2.1. Bat Samples

**Figure 1:**
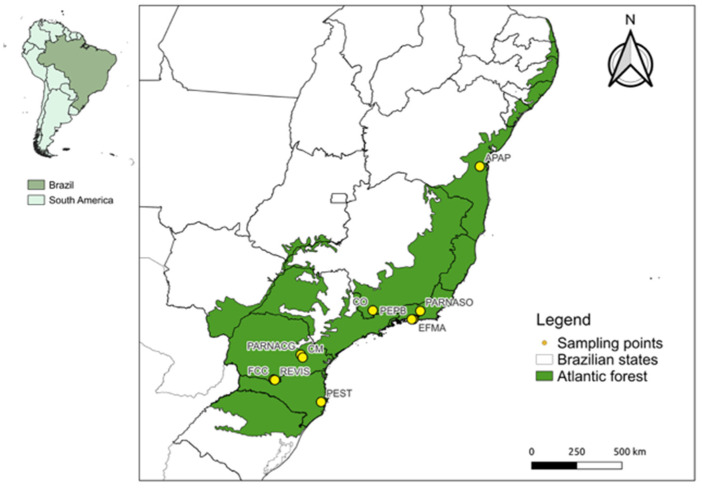
Atlantic Forest Biome (green) and sampling sites (points). Locality acronyms: APAP (Pratigi Protected Area); EFMA (Fiocruz Atlantic Forest Biological Station); PARNASO (Serra dos Órgãos National Park); PEPB (Pedra Branca State Park); FCC (Cerro Chato Farm); PARNACG (Campos Gerais National Park); REVIS (Campos de Palmas Wildlife Refuge); CM (Mariquinha Waterfall); CO (Conceição dos Ouros) and PEST (Serra do Tabuleiro State Park).

The present study analyzed 336 bat samples from the Atlantic Forest biome of the following five Brazilian states: Bahia (180); Rio de Janeiro (73); Paraná (40); Minas Gerais (22); and Santa Catarina (21). The captures occurred intermittently between December 2013 and September 2022, carried out on independent expeditions that included 10 sampling localities (Figure 1). The studied areas encompass different phytophysiognomies and conservation levels within the Atlantic Forest. Briefly, EFMA (Fiocruz Atlantic Forest Biological Station) and PEPB (Pedra Branca State Park) are remnants of dense rainforest surrounded by a matrix of urban areas. These locations are located within one of the most densely populated areas of the Rio de Janeiro metropolitan region, suffering from intense anthropic pressure. The locations of FCC (Cerro Chato Farm), CO (Conceição dos Ouros), CM (Mariquinha Waterfall) and REVIS (Campos de Palmas Wildlife Refuge) are composed of patches of rainforest and seasonal forest amidst a matrix formed by open fields, pastures, and agricultural areas with intense land use. Some of these areas are still under pressure due to deforestation, leading to the reduction and fragmentation of the remnants. Finally, the sampling locations of PARNASO (Serra dos Órgãos National Park), PARNACG (Campos Gerais National Park), PEST (Serra do Tabuleiro State Park), and APAP (Pratigi Protected Area) comprise protected areas formed by large remnants of dense rainforest with little anthropic disturbance.

The taxonomic identification of the individuals was made based on external and craniodental morphological characters and comparisons with specimens in museums and identification keys [20,21]. Bionomic data (body weight, forearm, tragus, foot, head-body, and tail lengths) and sex identification were obtained according to previous studies [21,22,23].

The captured bats were euthanized using a protocol of injectable anesthetics and analgesics approved by CEUA-Fiocruz. Samples of 0.3 to 3 mL of whole blood were collected from the animals by cardiac puncture and placed in a sterile plastic tube. Variations in volume were directly related to the animal’s body mass. After half an hour, the blood was centrifuged at 2700× *g* (Daiki Centrifuge, MyLabor^®^, Tokyo, Japan) for 20 min to separate the serum. Tubes containing between 0.1 and 1 mL of serum were stored at −20 °C until serological analysis.

Bats were grouped into four families (Phyllostomidae, Vespertilionidae, Molossidae, and Noctilionidae), in which 22 genera and 31 species were identified (Table 1). The Phyllostomidae family was the most representative, with 21 species distributed in eight subfamilies (Carolliinae, Stenodermatinae, Phyllostominae, Glyphonycterinae, Glossophaginae, Desmodontinae, Micronycterinae, and Rhinophyllinae). The Vespertilionidae family was represented by six species and two subfamilies (Vespertilioninae, Myotinae). The Molossidae family had one subfamily (Molossinae). The Noctilionidae family included only one genus, *Noctilio*, and two species, where one individual of the species *Noctilio leporinus* was collected. Regarding the trophic guilds, we observed frugivorous, omnivorous, insectivorous, hematophagous, carnivorous, nectarivorous, and piscivorous species among the studied bat species. Moreover, a similar number of males and females was observed in this investigation, with 173 males (51%), 154 females (46%), and nine individuals who remained unidentified in the field.

### 2.2. Ethical Statements

The procedures for sampling bats in this study were approved by the Animal Ethics Committee (CEUA) under process number P.42/12–1 (LW-81/12); P.62/11–3 (LW-68/12) and P.23/17.4 (LM-6/18) and by the Chico Mendes Institute for the Biodiversity Conservation (ICMBio/SISBIO) number 43065-1/26934-1 e number 17131-4.

### 2.3. Recombinant Nucleoproteins Expression

The expression and purification of ANDV, SEOV, XSV, and BRNV rNPs were performed in *Escherichia coli* as previously described [24]. Briefly, the cDNA encoding each of the studied proteins was cloned into the plasmid pET21a(+) to express the nucleoprotein with a six-histidine tag at its C-terminus (Genscript, Middlesex, NJ, USA). This construct was used to transform the BL21DE3 strain of *E. coli*, and the protein was expressed for 4 h with the addition of 1 mM IPTG. Cells were pelleted by centrifugation at 5000× *g* for 15 min at 4 °C and stored at −80 °C until use. The cell pellet was resuspended in a buffer containing 50 mM sodium phosphate (pH 6.0), 500 mM NaCl, 1 mM β-mercaptoethanol, 5% glycerol, 10 mM imidazole, and an EDTA-free protease inhibitor cocktail (Roche, Germany). Cell lysis was achieved by adding 1 mg/mL lysozyme and stirring at 4 °C for 30 min. Then, 20 μg/mL DNase and 2 mM MgCl_2_ were added, and the solution was incubated for another 30 min. The lysate was sonicated 15 times with alternating 15 s on and off cycles at 4 °C. After sonication, the lysate was centrifuged at 27,000× *g* for 20 min to obtain the protein pellet inclusion body. This pellet was solubilized in a buffer containing 50 mM sodium phosphate (pH 6.0), 500 mM NaCl, 1 mM β-mercaptoethanol, 5% glycerol, 10 mM imidazole, and 8 M urea, and stirred overnight at 4 °C. The clarified lysate was applied to a HisTrap HP column connected to an Äkta Purifier HPLC system. Proteins were eluted with a linear gradient of imidazole (10–500 mM) prepared in the same buffer, and the purified nucleoprotein was refolded by dialysis using a MWCO 3500 Da cut-off membrane and the same buffer composition overnight at 4 °C, with three buffer changes of 1 L each. Recombinant NPs (BRNV, XSV, ANDV, and SEOV) were obtained with high purity and yield, as analyzed by 15% SDS-PAGE, and a single band of approximately 50 kDa was observed. The refolding process of the rNPs was confirmed by fluorescence spectroscopy.

### 2.4. Serological Assay

Bat serum samples were evaluated for IgG antibodies against the rNPs of ANDV, SEOV, XSV, and BRNV as the specific antigen by enzyme-linked immunosorbent assay, as previously described and adapted from Padula et al. [14]. Briefly, to define a panel of negative, non-serum-reactive bat samples for hantavirus, we tested all the samples by indirect ELISA, at a dilution of 1:100, against the four rNPs hantavirus antigens and the streptavidin protein (Sigma-Aldrich, Brazil). In addition, monoclonal antibodies against ANDV and SEOV rNPs were used as positive controls. Briefly, 96-well, flat-bottomed MaxiSorp plates (Thermo Scientific Nunc, Rochester, NY, USA) were coated with 200 ng/well with one of the 5 antigens (rNP ANDV, SEOV, XSV, BRNV and Streptavidin) separately, in a carbonate–bicarbonate buffer at pH 9.6 and incubated overnight at 4 °C. After the incubation, the plates were washed three times with PBS–Tween-20 0.05% (PBS-T) and blocked by adding 200 µL/well of PBS-T-Skim milk 5% (PBS-block), for 1 h at 37 °C. After this stage, the plates were washed again, as described above, and serum samples (diluted 1:100 in PBS-block) were added in duplicate against each coated antigen and incubated for 1 h at 37 °C. Subsequently, the plate was washed six times with PBS-T. Then, 100 µL of antibody against bat IgG conjugated to horseradish peroxidase (HRP) (Bethyl Laboratories, Montgomery, TX, USA), diluted 1:1000 in PBS-block, was added to the samples and incubated for 1 h at 37 °C. After this step and six washes with PBS–T, we added 100 µL of the substrate tetramethylbenzidine (TMB) (Sigma-Aldrich, Brazil) in the dark and then added 50 µL per well of the stop solution (HCl-1N). Finally, the optical density was obtained from the absorbance of the ELISA reader (Molecular Devices/E-Max) measured at 450 nm. Based on these results, samples with similar absorbance among the five tested proteins were selected as a negative control with which to investigate serological reactivity in the panel of samples.

Using a methodology similar to that described above, all samples were tested against each rNP tested (rNP ANDV, SEOV, XSV, and BRNV) at a 1:400 dilution. To define reactive and non-reactive sera, we used the absorbance value of the negative control to establish a cut-off value defined as mean absorbance of the negative control plus 3 times its standard deviation. We then calculated the reactivity index (RI) of the samples as the ratio between the absorbance of each sample and the cut-off value for that protein. Samples with an RI greater than 1.05 were considered serum-reactive for the protein tested.

### 2.5. Absorption ELISA Using BRNV and ANDV

The absorption ELISAs were performed, as previously described [25,26], using BRNV and ANDV rNPs to demonstrate the cross-reactivity of antibodies between a bat-borne and rodent-borne rNP as antigens. Briefly, 96-microwell plates (NUNC-Maxisorp) were coated overnight with 2 µg/mL of the rNP ANDV or BRNV and then washed and blocked as previously described. First, sera were added to the plates coated with one of two tested rNPs at 1:100 titers and incubated for one hour at 37 °C. After incubation, sera were transferred to plates coated with the other rNPs (ANDV or BRNV) and incubated for one hour at 37 °C. Then, ELISA was finished, as described above, and the OD values against the rNPs ANDV and BRNV of the sera before and after absorption were compared.

### 2.6. Statistical Analysis

All statistical analyses were conducted using Prism 9.0 (GraphPad Software, Inc., San Diego, CA, USA). The one-sample Kolmogorov–Smirnoff test was used to determine whether a variable was normally distributed. Differences in frequencies of IgG responders to rNPs were evaluated using Fisher’s exact test. The Wilcoxon matched-pairs signed-rank test was used to compare reactivity indices against the rNPs. A two-sided *p*-value < 0.05 was considered significant.

## 3. Results

### 3.1. Evaluation of Serological Evidence of Hantavirus

**Figure 2:**
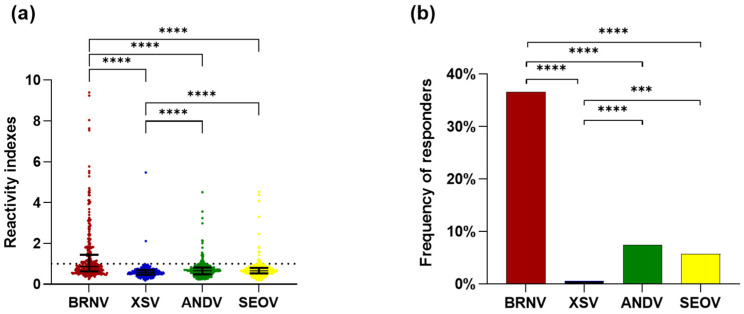
Evaluation of seroreactivity of Atlantic Forest bats against hantavirus nucleoproteins: (**a**) reactivity indexes against the rNPs of BRNV (red dots), XSV (blue dots), ANDV (green dots), and SEOV (yellow dots). Each point represents the RI of one sample for each nucleoprotein and the lines represent the median and interquartile range. The dashed line indicates the threshold for positivity (1.05). RIs were compared between rNPs by the Wilcoxon matched-pairs signed rank test, with an asterisk indicating significant differences: *p* < 0.00001 (****); and (**b**) frequencies of responders to hantavirus nucleoproteins. The frequencies of responders to the rNPs of BRNV (red bar), XSV (blue bar), ANDV (green bar), and SEOV (yellow bar) were compared by Fisher’s test. Significant differences are indicated by an asterisk *: *p* < 0.001 (***), *p* < 0.00001 (****).

We evaluated the IgG response of 336 serum samples from bats collected in the Atlantic Forest biome, using ELISA at a dilution of 1:400, against four recombinant NPs of ANDV, SEOV, XSV, and BRNV. First, we observed that the RIs ranged from 0.18 to 9.4. Based on the Wilcoxon matched-pairs signed-rank test, the RIs against BRNV rNP (median: 0.869, interquartile range: 0.627–1.447) were statistically higher (*p* < 0.0001) than the RIs against the rNPs of XSV (median: 0.577, interquartile range: 0.47–0.693), ANDV (median: 0.665, interquartile range: 0.489–0.821), and SEOV (median: 0.656, interquartile range: 0.53–0.804). Moreover, the reactivity indexes against the rNP of XSV were lower than those against ANDV and SEOV (*p* < 0.0001; Figure 2a).

Regarding the frequency of responders to each studied rNP, BRNV was recognized by the antibodies of 123 tested bat samples, presenting a frequency of responders of 36.6%, which was statistically higher (*p* < 0.0001) than the frequency of responders of ANDV (7.4%, *n* = 25), SEOV (5.7%, *n* = 19), and XSV rNPs (0.6%, *n* = 2). The frequency of responders for XSV was also lower than that for ANDV (*p* < 0.0001) and SEOV (*p* = 0.0002), while the rNPs of ANDV and SEOV were recognized by a similar number of samples (*p* = 0.4359) with similar reactivity indexes (*p* = 0.4979; Figure 2b). Moreover, the RIs against streptavidin were similar to the RIs against the *E. coli* cell lysate.

In addition, considering the reactivity at the serum dilution of 1:400, 139 samples were reactive for at least one of the rNPs studied. Among these, 100 samples were exclusively reactive to the BRNV rNP, nine to the ANDV rNP, three to the SEOV rNP, and one sample to the XSV rNP. In addition, nine samples were simultaneously reactive to BRNV and ANDV, nine to BRNV and SEOV, one to BRNV and XSV, two to ANDV and SEOV, and four samples were cross-reactive to BRNV, SEOV, and ANDV. Remarkably, all 139 samples were reactive to BRNV at the dilution of 1:100. These data, allied to the higher frequency of responders and magnitude of response (RI) to the BRNV NP corroborates that this protein is a better antigen with which to investigate serological evidence of hantaviruses in bats from the Brazilian Atlantic Forest.

### 3.2. Evaluation of Cross-Reactivity Between BRNV and ANDV

**Figure 3:**
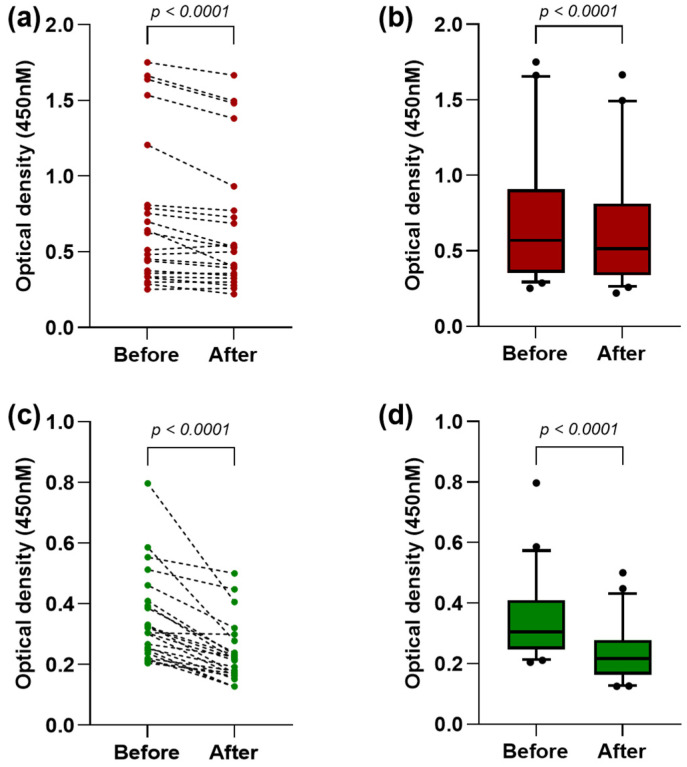
Evaluation of antibody cross-reactivity between hantavirus nucleoproteins: (**a**) optical densities (OD) against BRNV rNP before and after the absorption of antibodies to the ANDV rNP. Each red point represents the OD of one sample, and the dashed lines connect the OD before and after the absorption; (**b**) OD against the BRNV rNP before and after the absorption to the ANDV rNP, represented by red boxes and whiskers (10–90 percentile), with outliers indicated by black points in the last graph; (**c**) OD against the ANDV rNP before and after the absorption of antibodies to the BRNV rNP. Each green point represents the OD of one sample, and the dashed lines connect the OD before and after the absorption; and (**d**) OD against the ANDV rNP before and after absorption to the BRNV rNP, represented by green boxes and whiskers (10–90 percentile), with outliers indicated by black points in the last graph. The Wilcoxon matched-pairs signed-rank test was used to compare the mean OD values before and after the absorption.

The absorption ELISA was performed using samples that reacted with the ANDV rNP, to verify the cross-reactivity between the NPs. As shown in Figure 3a, after the absorption of antibodies to the ANDV rNP, the reactivity to the BRNV rNP was significantly reduced (*p* < 0.0001), with the mean OD changing from 0.74 (±0.49) to 0.66 (±0.45) (Figure 3b). Likewise, the reactivity reduction was more evident to ANDV after the absorption of antibodies to the BRNV rNP (Figure 3c), with the mean OD being reduced from 0.35 (±0.15) to 0.23 (±0.1) (*p* < 0.0001) (Figure 3d).

### 3.3. Eco-Epidemiological Data of Seroreactive Bats from the Brazilian Atlantic Forest

**Figure 4:**
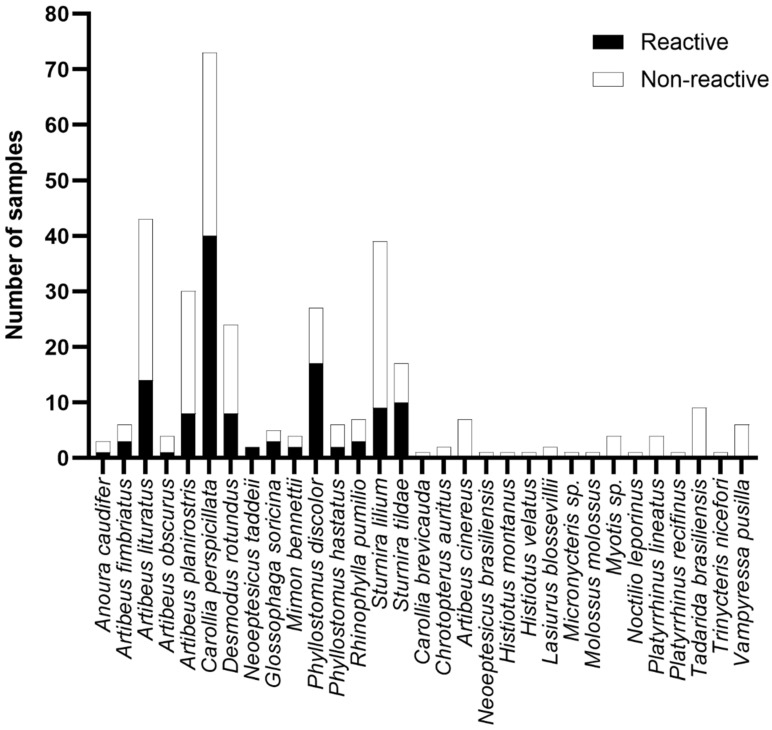
Seroreactivity against BRNV among the bats from the Brazilian Atlantic Forest species analyzed. Bars indicate the number of samples tested. Black bars indicate reactive samples and white bars indicate non-reactive samples. The name of the species is given on the *y*-axis.

Based on our data supporting the BRNV rNP as a better antigen for investigating the serologic evidence of hantavirus in bats, we explored the eco-epidemiological data of bats seroreactive to this protein. First, we observed seroreactive samples from 15 different species belonging to 10 genera (*Anoura*, *Artibeus, Carollia, Desmodus, Neoeptesicus*, *Glossophaga*, *Mimon*, *Phyllostomus, Rhinophylla*, and *Sturnira*) grouped in two families (Phyllostomidae and Vespertilionidae). As shown in Figure 4, the frequency of positive samples among the seroreactive species ranged from 27% to 100%; however, the difference in the number of samples of the studied species, which ranged from 1 to 73, probably biased these frequencies. Remarkably, we observed no reactivity in 16 of the studied species against BRNV or the other rNPs (*Carollia brevicauda*, *Chrotopterus auritus*, *Artibeus cinereus*, *Neoeptesicus brasiliensis*, *Histiotus montanus, Histiotus velatus, Lasiurus blossevillii*, *Micronycteris* sp., *Molossus molossus, Myotis* sp., *Noctilio leporinus, Platyrrhinus lineatus*, *Platyrrhinus recifinus*, *Tadarida brasiliensis*, *Trinycteris nicefori*, *Vampyressa pusilla)*; however, the limited number of samples of these species, ranging from one to nine, needs to be highlighted (Figure 4).

Likewise, the number of samples collected in the study sites varied considerably; some had less than 10 individuals, where the frequency of positive samples ranged from 6% to 43%, considering the number of samples analyzed. Therefore, it was not possible to observe significant seroreactivity differences among the studied areas that encompass distinct levels of conservation. However, it is worth noting that one of the highest prevalence rates was observed in EFMA (32%), which is considered a highly impacted area that is also close to densely populated areas of the Rio de Janeiro metropolitan region.

**Figure 5:**
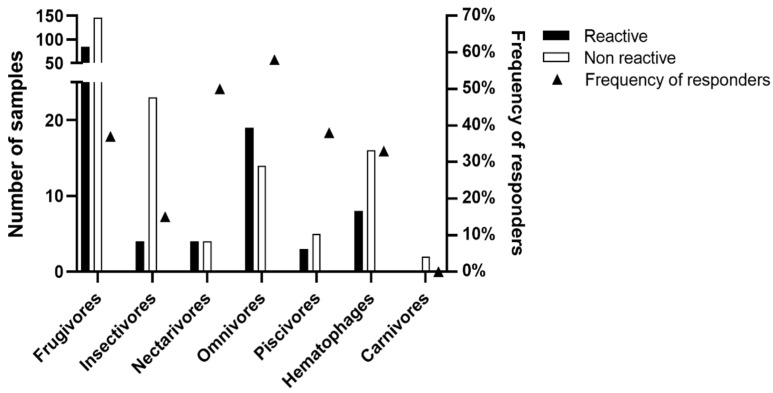
Seroreactivity to BRNV recombinant nucleoprotein among bat trophic guilds in Brazilian Atlantic Forest. The number of seroreactive samples and the total number of samples for each trophic guild are indicated by black and white bars, respectively, on the left *y*-axis. The triangles denote the frequency (%) of seroreactive samples within each trophic guild on the right *y*-axis.

As shown in Figure 5, of the seven trophic guilds described in the studied bat populations, we observed seroreactivity to the BRNV rNP in six guilds, with 85 seroreactive frugivores, four insectivores, four nectarivores, 19 omnivores, three piscivores, and eight hematophages. In our study, only carnivores did not present any reactive samples to the BRNV NP. As shown in Figure 4, the number of seroreactive samples is not necessarily related to the frequency of responders by trophic guild. Here, the frequencies of responders among frugivores, insectivores, nectarivores, omnivores, piscivores, and hematophages were 37%, 15%, 50%, 58%, 38% and 33%, respectively. The differences in the number of samples associated within each trophic guild, ranging from two (carnivores) to 231 (frugivores), prevent adequate statistical analysis and conclusive definitions of the relationship between trophic guilds and hantavirus seroreactivity. In addition, we observed no differences related to sex on the reactivity against the BRNV NP. Of the 123 seroreactive bats, 59 (48%) were males, 60 (49%) were females, and four were unidentified samples. Based on the Mann–Whitney test, there were no differences in the RIs of males (mean 2.7 ± 1.9) and females (mean 2.3 ± 1.5) (*p* = 0.4092).

## 4. Discussion

The study of hantaviruses has predominantly focused on rodent hosts, leaving a significant knowledge gap regarding bat-associated hantaviruses, especially in neotropical regions such as the Brazilian Atlantic Forest [17,19]. Our research aimed to address this gap by evaluating the seroprevalence and cross-reactivity of bat samples with four hantavirus nucleoproteins. The results show a remarkable seroprevalence of antibodies against the BRNV rNP, suggesting that bats in this biome may harbor hantaviruses antigenically related to BRNV. This discovery underscores the importance of expanding hantavirus surveillance beyond rodents to include bat populations, which could be critical to understanding the full epidemiologic landscape of hantavirus infections and developing more effective diagnostic tools [10].

In this study, 336 bat blood or serum samples were tested for two bat-borne hantaviruses rNPs (BRNV and XSV) and two rodent-borne hantaviruses rNPs (ANDV and SEOV). In this serological evaluation, we observed a high seroprevalence for the BRNV rNP, with about 37% of the samples reactive to this protein, while less than 10% of the samples recognized rodent-borne hantaviruses rNPs, and less than 1% recognized the XSV rNP. Interestingly, we observed that the samples that recognized rodent-borne hantaviruses also cross-reacted with BRNV, even at 1:100 dilution, suggesting that these bats were naturally exposed to an unknown hantavirus that is antigenically closely related to BRNV. The evaluation of the reactivity of bat samples against bat-borne and rodent-borne hantaviruses was previously proposed by Xu and collaborators [16], who evaluated the reactivity of 709 bats from five Chinese provinces against the rNPs of the bat-borne hantavirus LAIV and XSV, and the rodent-borne hantavirus SEOV. The authors observed a total serum reactivity rate of 18.5% (131/709) and demonstrated cross-reactivity between XSV/SEOV and LAIV/XSV, but not LAIV/SEOV, indicating that XSV is antigenically closer to human-infecting hantaviruses [16]. However, studies regarding bat-borne hantaviruses in America remain limited [17,19].

To date, studies focused on investigating the serologic evidence of bat-borne hantaviruses in Brazil have used only rodent-borne rNPs [17,18,19,27,28]. In 2018, Sabino and collaborators analyzed 53 bat samples from the southeastern Atlantic Forest biome of Brazil by ELISA against Araraquara virus (ARAV) rNPs [18]. ARAV is a Brazilian viral variant of ANDV related to *Necromys lasiurus* and, like ANDV, is associated with HPS [18]. Sabino et al. observed seroreactivity for ARAV rNP in 17% of the studied samples, reporting antibodies in *Desmodus rotundus* (*n* = 3), *Artibeus lituratus* (*n* = 1), *Artibeus obscurus* (*n* = 1), *Artibeus planirostris* (*n* = 1), and *Carollia perspicillata* (*n* = 1). Our study is in accordance with Sabino’s study; we observed reactivity in all listed species. Moreover, we also detected reactivity to BRNV in *Anoura caudifer*, *Artibeus fimbriatus*, *Neoeptesicus taddeii*, *Glossophaga soricina*, *Mimon bennettii*, *Phyllostomus discolor*, *Phyllostomus hastatus*, *Rhinophylla pumilio*, *Sturnira lilium*, and *Sturnira tildae*, species associated with different trophic guilds, which may be correlated to a diversity of routes of viral transmission and spread in their ecological niches. Notably, the higher number of samples reactive to the BRNV rNP suggests that this protein is antigenically closer to the bat-borne hantaviruses in Brazil and supports its choice as a more sensitive antigen for the serological screening of hantavirus in Brazilian bats. However, further studies are needed to evaluate its potential in the investigation of other Brazilian biomes and throughout the Americas.

Recently, our group published in silico data suggesting that bat-borne hantavirus NPs are weakly conserved with ANDV and its variants [10]. In this context, this was the first evaluation of the seroreactivity of Brazilian bat samples using bat-borne hantaviruses rNPs as ELISA antigens. Remarkably, our data are in agreement with the in silico findings [10], as we observed a statistically higher frequency of responders and reactivity against BRNV rNP; a minimal cross-reactivity with XSV rNP, another bat-borne hantavirus; and ANDV and SEOV, rodent-borne hantaviruses. Furthermore, the minimal cross-reactivity observed corroborates not only our in silico results, but also the study by Xu and collaborators [16], suggesting that the Brazilian hantavirus is antigenically close to BRNV and distant from the southeast Asian bat-borne hantavirus. This suggestion is supported by the fact that bats described as seroreactive to XSV belong to the family Hipposideridae, known as “roundleaf bats”, which do not occur in the Americas [29].

BRNV was first described in the Czech Republic in the species *Nyctalus noctula*, a vespertilionid bat found in Europe and Central Asia [30], where no serologic testing of bat samples has been performed. Dafalla et al. [31], using RT-PCR on bat tissue samples from Germany (*n* = 245), Austria (*n* = 207), and Poland (*n* = 20), found the presence of the BRNV virus in three (1.2%), one (0.5%) and three (20%) of the samples, respectively. All the positive samples were from *N. noctula* bats, suggesting a high host specificity [30,31,32]. Conversely, despite our data reporting the high seroreactivity of Brazilian bat serum samples to BRNV rNP, only 3.3% (*n* = 11) of the tested samples belonged to the family Vespertilionidae; among them, only two samples, described as *N. taddeii*, were seroreactive. However, based on the aggregation habits of bats, we cannot exclude the possibility of Phyllostomidae bats being exposed to Brazilian bat-borne hantaviruses through interspecific contact. In fact, recently, partial sequences of two hantaviruses recovered from *C. perspicillata* captured in the North and Northeastern regions of Brazil were described [33,34]. This possibility underscores the urgency of the genomic detection and characterization of South American bat-borne hantaviruses to understand the correlations between RT-PCR and antibody reactivity and to obtain a true picture of hantaviruses in neotropical bats.

Our findings highlight the importance of expanding hantavirus surveillance to include bat populations, especially in biodiverse regions such as the Brazilian Atlantic Forest. The high seroprevalence of antibodies to the BRNV rNP in bats suggest the presence of hantaviruses closely related to BRNV, which may have significant public health implications, considering the potential risk of occasional spillover to humans and other animals. Furthermore, the observed cross-reactivity between BRNV and rodent-borne hantavirus nucleoproteins highlights the need for specific antigens in serological tests to improve the accuracy of hantavirus detection in bats. Future research should focus on the ecological dynamics of hantavirus infections within bat populations, the potential for human spillover, and the genomic characterization of American bat-borne hantaviruses. This study provides a critical foundation for the development of more effective diagnostic tools and surveillance strategies for bat-borne hantaviruses on the American continent.

## Figures and Tables

**Table 1:** Taxonomic (family and species) and geographic (state, locality) sampling of bats analyzed. State acronyms: BA (Bahia); RJ (Rio de Janeiro); PR (Paraná); MG (Minas Gerais); SC (Santa Catarina). Locality acronyms: APAP (Pratigi Protected Area); EFMA (Fiocruz Atlantic Forest Biological Station); PARNASO (Serra dos Órgãos National Park); PEPB (Pedra Branca State Park); FCC (Cerro Chato Farm); PARNACG (Campos Gerais National Park); REVIS (Campos de Palmas Wildlife Refuge); CM (Mariquinha Waterfall); CO (Conceição dos Ouros) and PEST (Serra do Tabuleiro State Park).

		Individuals Per Locality									
		BA	RJ			PR				MG	SC
Family	Species	APAP	EFMA	PARNASO	PEPB	FCC	PARNACG	REVIS	CM	CO	PEST
Phyllostomidae	*Carollia perspicillata*	52	4	4	3					4	7
	*Carollia brevicauda*	1									
	*Artibeus lituratus*	24	4	7	2					2	4
	*Artibeus cinereus*	7									
	*Artibeus planirostris*	27		2	1					1	
	*Artibeus fimbriatus*	2	1								2
	*Artibeus obscurus*	1			3						
	*Sturnira lilium*	9	4	5		1	2	2	2	10	4
	*Sturnira tildae*	6	1	9						1	
	*Platyrrhinus lineatus*	2			1					1	
	*Platyrrhinus recifinus*				1						
	*Vampyressa pusilla*		2		1						3
	*Phyllostomus discolor*	27									
	*Phyllostomus hastatus*	6									
	*Mimon bennettii*								4		
	*Chrotopterus auritus*			1			1				
	*Trinycteris nicefori*	1									
	*Glossophaga soricina*	4	1							2	
	*Anoura caudifer*	1		1							1
	*Desmodus rotundus*	1	10		3		3		7		
	*Micronycteris* sp.		1								
	*Rhinophylla pumilio*	7									
Vespertilionidae	*Neoeptesicus taddeii*					2					
	*Neoeptesicus brasiliensis*								1		
	*Histiotus montanus*					1					
	*Histiotus velatus*					1					
	*Lasiurus blossevillii*							1	1		
	*Myotis* sp.	1						1	1	1	
Molossidae	*Molossus molossus*				1						
	*Tadarida brasiliensis*							9			
Noctilionidae	*Noctilio leporinus*	1									
	Total	180	28	29	16	5	6	13	16	22	21

## Data Availability

No new data were created. All data presented in this study are summarized in the paper. The detailed data of this study are available on request from the corresponding author.
